# Acetylation of the Proto-Oncogene EVI1 Abrogates Bcl-xL Promoter Binding and Induces Apoptosis

**DOI:** 10.1371/journal.pone.0025370

**Published:** 2011-09-28

**Authors:** Anjan Kumar Pradhan, Alok Das Mohapatra, Kasturi Bala Nayak, Soumen Chakraborty

**Affiliations:** 1 Department of Gene Function and Regulation, Institute of Life Sciences, Bhubaneswar, Orissa, India; 2 Department of Infectious Disease Biology, Institute of Life Sciences, Bhubaneswar, Orissa, India; University of Hong Kong, Hong Kong

## Abstract

EVI1 (Ecotropic Viral Integration site I), which was originally identified as a myeloid transforming gene by means of retroviral insertional mutagenesis in mouse leukemia, encodes a nuclear DNA binding zinc finger protein. The presence of zinc fingers that are able to bind to specific sequences of DNA suggests that EVI1 is a transcriptional regulator; however, except a few, target genes of EVI1 are poorly functionally identified thus far. In this study we provide evidence that EVI1 directly induces the expression of Bcl-xL through the first set of zinc finger and thereby inhibits apoptosis. ChIP analysis showed that EVI1 binds to the Bcl-xL promoter in HT-29 cells, a colon carcinoma cell line, which expresses EVI1. The observation is also supported by the fact that EVI1 siRNA treated HT-29 cells, shows a down regulation of Bcl-xL expression and that over expression of EVI1 results in the induction of the Bcl-xL reporter construct. A set of EVI1 positive chronic myeloid leukemia (CML) samples also showed higher Bcl-xL expression with respect to EVI1 negative samples. Interestingly, co-expression of EVI1 with wild type, but not with dominant-negative form of PCAF, abolishes the effect of EVI1 on Bcl-xL, indicating that acetylation of EVI1 abrogates its ability not only to bind Bcl-xL promoter but also alleviate Bcl-xL activity. Finally we have shown that EVI1 expression regulates apoptosis in HT-29 cells, which is abrogated when HT-29 cells are transfected with EVI1 siRNA or PCAF. The result for the first time shows a direct pathway by which EVI1 can protect cells from apoptosis and also demonstrates that the pathway can be reversed when EVI1 is acetylated.

## Introduction

One of the genes associated with both murine and human myeloid leukemia is EVI1 [1], [2]. Over expression and aberrant expression of EVI1 was shown to be associated with most forms of human leukemia, as a consequence of chromosomal rearrangements involving 3q26.2, where the gene is mapped [3] and also without cytogenetically detectable rearrangements of the EVI1 locus as a result of unknown mechanism [2], [4]. Overall up regulation of EVI1 has been shown in 30% of advanced CML patients, 8–10% of MDS –AML patients and 7.8% of de novo AML cases [5]–[7]. Also studies indicate that EVI1 may be over expressed in a subset of human colon cancers, and that EVI1 might affect disease progression and/or sensitivity to chemotherapy [8]. The protein is highly conserved through evolution and encodes a repressor and an activator domain with two sets of zinc finger motifs [1], [3], [9], [10]. Both zinc finger domains of EVI1 recognize and bind to specific DNA consensus sequence *in vitro* and *in vivo*, of which some are characterized and some are not functionally characterized [3], [11], [12]. Some known functionally relevant targets of EVI1 includes Pbx1, calreticulin and GATA2 which are involved in calcium metabolism, maintenance of adequate number of hematopoietic stem cells and self renewal of hematopoietic stem cells [11], [13], [14]. However, the exact mechanism by which EVI1 promotes cell proliferation and blocks apoptosis still remains unclear.

Apoptosis is a critical process that is deregulated in oncogenesis [15]. Bcl-2 and Bcl-xL are anti-apoptotic paralogues that inhibit apoptosis which is elicited by a wide variety of stimuli, and play critical roles in cancer development and resistance to treatment whereas BAD, BAX, BID and PUMA enhance DNA damage induced apoptosis [16]. Subtle changes in the cellular levels of Bcl-xL expression can have dramatic phenotypes. Recently it was shown that HIF-1α can directly up regulate Bcl-xL gene transcription and can protect prostate cancer cells from apoptosis [17]. Inducible expression of EVI1 or MDS1/EVI1 in the human myelomonocytic cell line U937T lead to cell cycle arrest and massive apoptosis upon exposure to differentiation stimuli, thereby recapitulating salient aspects of the biology of MDS *in vitro* [18]. EVI1 activates PI3K/AKT signaling and suppresses TGF-β mediated apoptosis [8]. This property of EVI1 appears likely to be integral to its oncogenic potential, since loss of response to apoptotic signals is one of the signs of transformation.

Posttranslational modification of proteins is a hallmark of signal transduction and it allows existing proteins to react rapidly to extra cellular events cascading to a total cellular response. These modifications are tightly controlled in the cell because they are involved in vital processes such as cell cycle progression, differentiation and apoptosis. It was shown earlier that interaction of EVI1 with different co-regulators could result in periodic, reversible acetylation and deacetylation of EVI1 and assembly of acetylated EVI1 in nuclear speckles [19]. The nuclear speckles are associated with cellular functions such as DNA replication, gene transcription and regulation of apoptosis. Evasion of apoptosis has been observed in both EVI1 positive hematopoietic and epithelial cells and suggests that EVI1 is a survival factor [8], [20], [21], however none of them are mutually exclusive and additional mechanisms mediating the survival promoting effects of EVI1 may also exist. Here we show by several approaches that the first set of zinc finger of EVI1 directly binds to the Bcl-xL promoter sequence both *in vitro* and *in vivo* and directly modulates its function by activating the anti-apoptotic gene and eventually blocks apoptosis. However PCAF acetylated EVI1 reverses the function and renders the cell towards apoptosis. These results point to a novel mechanism that can be therapeutically utilized directly to prevent EVI1 mediated block in apoptosis.

## Materials and Methods

Cell Culture and Plasmids- The human embryonic kidney cell line 293T, mouse fibroblast cell line NIH3T3 and human colon cancer cell line HT-29 used in this study were cultured in Dulbecco's modified minimum essential medium supplemented with 10% newborn calf serum (PAN Biotech, GmbH). Flag-EVI1-wt was constructed by PCR cloning in EcoRI and SalI site of Flag-pCMV vector (Sigma, USA) while pGL2 Bcl-xL (Bcl-xL promoter in pGL2 vector) was a kind gift from Dr. Nunez, Michigan University, USA. The Bcl-xL mutated promoter (Bcl-xL (Mut)) i.e. without the EVI1 binding site was cloned in the luciferase vector. EVI1 without either 1^st^ (EVI1- Δ 24 to 239, will be called as flag-EVI1-ΔA) or 2^nd^ (EVI1- Δ 735 to 812, will be called as EVI1-ΔD) set of zinc fingers were cloned by standard PCR method in EcoRI and SalI site Flag-pCMV vector. All the constructs were verified by sequencing and expression was checked by Western blotting.

**Figure 1:**
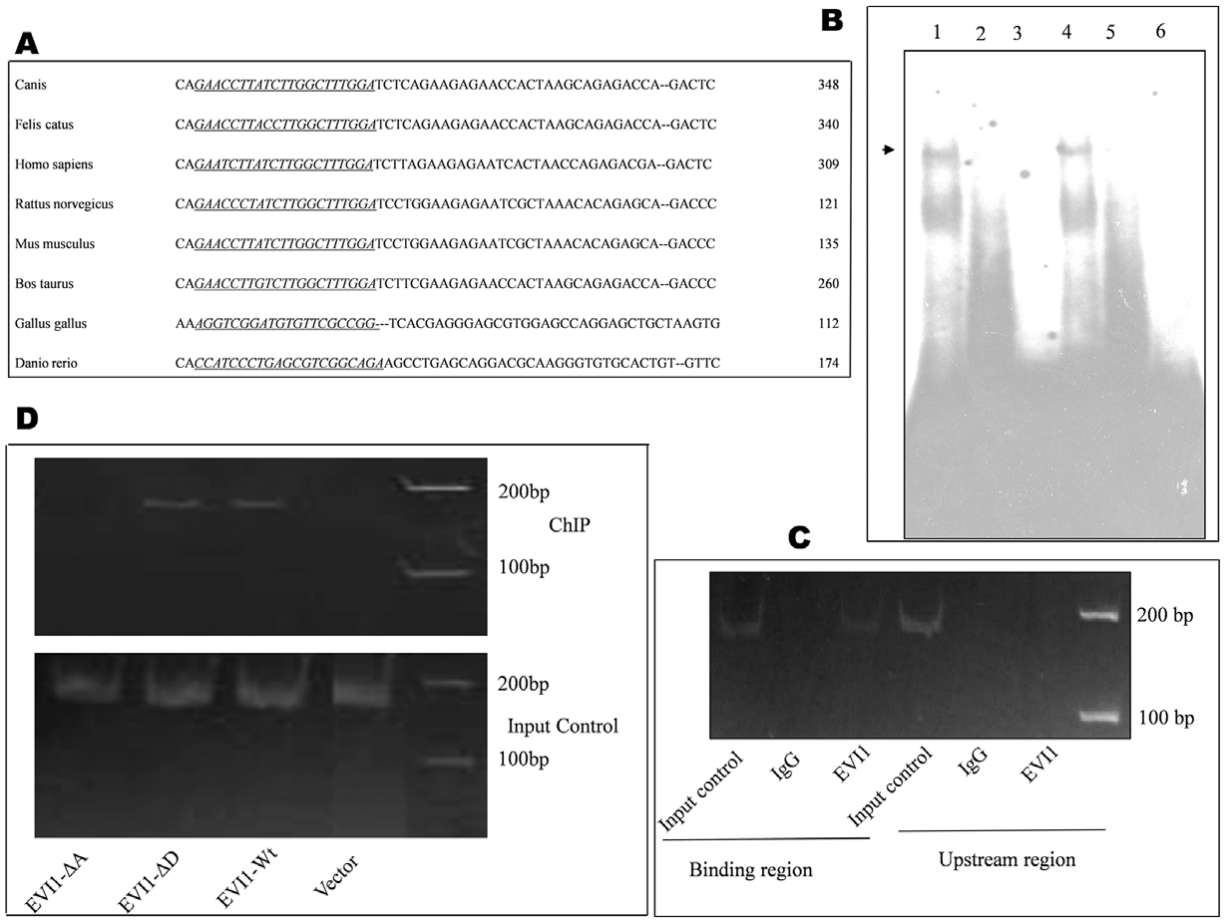
EVI1 binds to Bcl-xL promoter element. A. A multiple sequence alignment of parts of Bcl-xL 2^nd^ exon from different species are shown and the binding site is underlined. Genbank accession numbers of each of Bcl-xL sequence from different species that were used in the alignment analysis are as follows, *Canis familiaris*: NM_001003072.1, *Homo sapiens*: NM_138578.1, *Rattus rattus*: NM_001033670.1, *Gallus gallus*: NM_001025304.1, *Danio rerio*: NM_131807.1, *Felis catus*: NM_001009228.1, *Bos taurus*: NM_001077486.2 and *Mus musculus*: NM_009743.4. B. Nuclear extracts from EVI1 transfected 293T cells were incubated with a radiolabelled, double-stranded oligo designed from the EVI1 binding site in Bcl-xL promoter. EVI1 binds to Bcl-xL (lane 1) as shown by an arrow whereas a mutated probe failed to show any binding (lane 2). Lane 3 shows only the empty vector transfected cells and lane 6 shows the probe without any nuclear lysate. Increasing amount of specific cold competitor at 30× molar excess of radiolabelled probe (lane 4) failed to inhibit the binding of EVI1 to Bcl-xL promoter however a cold competitor at 50× molar excess of radiolabelled probe (lane 5) inhibited binding of EVI1 to Bcl-xL promoter. C. ChIP analysis shows the occupancy of EVI1 on the Bcl-xL promoter *in vivo*. After cross linking and chromatin fractionations DNA protein complexes were incubated with EVI1 antibody and the complex were processed as per the manufacturer's instructions (Imgenex India Pvt. Ltd.). Eluted DNA samples were then analyzed by PCR using specific primers flanking the EVI1 binding region. A band of 170 bp was observed only in HT-29 cells (lane 3) and no band was observed with IgG (lane 2) or with primers taken from the upstream region (lane 6). Marker (M) and the input controls for both regions are as shown (lane 1 and lane 4). D. ChIP was performed on 293T cells transfected with empty vector or EVI1 construct. For this assay flag-CMV-vector, flag-EVI1 wild type (Wt), flag- EVI1-ΔA (EVI1-Δ24 to 239) and flag-EVI1-ΔD (EVI1-Δ735 to 812) constructs were transfected separately in 293T cells. After processing as described above PCR showed an amplification of 170 bp when cells were transfected with EVI1 and EVI1-ΔD where as no amplification was observed in vector transfected cells or EVI1-ΔA transfected cells. Marker and the input control are as shown.

Electrophoretic Mobility Shift Assay (EMSA) - Sequences of the Bcl-xL promoter oligonucleotide probes that were used include Bcl-xL, 5′-CAGAATCTTATCTTGGCTTTGG-3′ and the probe with mutation in the binding site is: Bcl-xL-mut, 5′-CAGAATCTAATCATGGCTTTGG-3′. For each probe, complementary strands were synthesized, and equimolar concentrations of complementary strands were annealed for use in EMSA. Wild-type and mutated probes were labeled using γ^32^P-ATP with T4 poly nucleotide kinase (Fermentas, Germany). For competition assays, 30× and 50× unlabeled wild-type probes were used. The human embryonic kidney cell line, 293T were harvested after transfection with different constructs as mentioned in Figure 1B and 3A. Nuclear extracts were prepared, and 10 µg of each was incubated with 100 pmol of labeled probe in a 10-µl reaction mixture (with 0.5 µg of poly dI∶ dC) (Sigma, USA) for 30 min at room temperature. The mixture was electrophoresed at 4°C on a 6% non-denaturing PAGE for 3 h and signals were detected by autoradiography.

Chromatin Immunoprecipitation- ChIP assay was carried out in both HT-29 cells and 293T cells by using the Quik ChIP kit (Imgenex India Pvt. Ltd, India). Briefly, as per the manufacturer's instructions only HT-29 cells or transfected 293T cells were cross linked with 1% formaldehyde for 10 minutes. Cells were then lysed, and nuclei were pelleted. The extract was sonicated, supernatants were collected and treated with sheared salmon sperm DNA and protein G agarose beads (Sigma, USA). Immunoprecipitation was performed overnight at 4°C with 3 µg of anti EVI1 antibody (Santa Cruz, USA) followed by protein G agarose beads (Sigma, USA) and control IgG. Precipitates were washed and extracted with 1% SDS and 0.1 M NaHCO_3_. Eluates were pooled, heated and the purified DNA fragments were used as template for PCR. The promoter-specific primers flanking the binding site used were: Bcl-xL-F-AGGGTAAATGGCATGCATATTAA and Bcl-xL-R-TTATAATAGGGATGGGCTCAACCA (amplicon size-170 bp). We used a pair of non-specific primers (EVI1-W) which were selected from a region that is approximately 1.27 kb upstream of the binding site, i.e., Bcl-xL-upstream-F-CTCTCAGGAAGGTCTAGGGGGCG and Bcl-xL-upstream-R-CGCCCCCGGGAGGGCTCCGGGGC (amplicon size-170 bp). The same procedure was followed with 293T cells transfected with empty Flag-pCMV vector, flag-EVI1-wt, flag-EVI1-ΔA and flag-EVI1-ΔD and immunoprecipitated with anti-flag antibody (Sigma, USA).

Si-RNA and Real-time Quantitative PCR- To knockdown the expression of EVI1, specific EVI1 siRNA and its negative control were transfected into HT-29 cells using the Icafectin siRNA transfection reagent (Eurogentec, Belgium). Total RNA was extracted from HT-29 cells, EVI1 siRNA transfected HT-29 cells and siRNA negative control transfected HT-29 cells by using the TRIzol reagent (Invitrogen, CA). The siRNA sequence that we used was 5′-3′- CCA-CGA-AGA-ACG-GCA-AUA-U99. Complementary cDNA was made by first strand cDNA synthesis kit (Fermentas, Germany). PCR primers were designed from the Bcl-xL mRNA reference sequence, NM_138578.1, which were as follows: Bcl-xL forward-GCCACTTACCTGAATGACCAC, Bcl-xL-reverse-TGGATGGTCAGTGTCTGGT (amplicon size-210 bp). The Real-time PCR (RQ-PCR) master mix containing SYBR Green (Eurogentec, Belgium) was used for real-time PCR on Opticon 2.0 (MJ Research), and the data were recorded and analyzed. Fold change of target genes (relative to β-actin) was defined by 2^−ΔΔCt^, where ΔΔCt = (Ct Bcl-xL−Ct β-actin for EVI1 positive HT-29 or 293T cells)−(Ct Bcl-xL−Ct β-actin for EVI1 negative HT-29 or 293T cells). Also RQ-PCR was used to analyze the Bcl-xL expression in 293T cells transfected with CMV vector control and flag-EVI1-wt construct. The same procedure was followed with CML patient's samples and is represented in terms of relative Ct values. P value was calculated by using graph-pad prism software, version 4.0.

Western Blot Analysis- 293T cells express the SV40 large T antigen and were used for transfection assays by the calcium phosphate precipitation method. After 48 h of transfection with flag-EVI1-wt only and flag-EVI1-wt with either PCAF-wt or PCAF-dominant negative (DN), cells were lysed by NP-40 based lysis buffer (Tris-25 mM, NaCl-50 mM, NP-40-1%). Total protein was resolved by SDS-polyacrylamide (Sigma, USA) gel electrophoresis and was electro blotted to polyvinylidene difluoride membrane (Amersham Biosciences, USA). After blocking the membrane with 5% nonfat milk (Biorad, USA) it was incubated with different primary and secondary antibodies at room temperature for 1 h and 40 minutes respectively. Signals were detected by exposure to X-ray films after treatment with the enhanced chemiluminescence (GE healthcare, USA). The primary antibodies that were used are: Bcl-xL from Imgenex India Pvt. Ltd., anti acetyl lysine antibody from cell signaling technology, USA, anti-flag antibody from Sigma Aldrich Pvt. Ltd., USA and secondary antibodies were from Santa Cruz Biotechnology, Santa Cruz, CA. Western blot analysis was used to detect the presence/absence of EVI1 in HT-29 cells and also HT-29 cells transfected with EVI1 siRNA and its negative control.

Reporter Gene Assay-The reporter construct pGL2 Bcl-xL, pGL2 Bcl-xL (mut) and the pRL-TK plasmid (Promega, USA) containing the Bcl-xL promoter in pGL2, promoter lacking the EVI1 binding site and renilla luciferase gene as internal control respectively were used in dual reporter gene assay for studying EVI1 dependent gene expression. 293T and NIH3T3 cells were transfected with different constructs (mentioned in figure) by using superfectamine (Qiagen, CA). Four hrs after transfection, the medium was replaced by fresh medium containing serum. Twenty four hrs after transfection, cells were treated with the lysis buffer with three cycle of cell freezing and thawing. The samples were immediately centrifuged, and luciferase activity was determined by using Luminometer (GLOMAX 20/20, Promega, USA). All measurements were done in triplicates, and the assays were repeated three times in both 293T and NIH3T3 cells. The PCAF siRNA used in this study was purchased from Sigma-Aldrich pvt. Ltd, USA. The siRNA sequence that we used was 5′-3′- CUC-UAA-UCC-UCA-CUC-AUU-U(dT)(dT). P value was calculated by using graph-pad prism software, version 4.0.

Apoptosis Study- HT-29 cells were transfected with the EVI1 specific siRNA, PCAF and PCAF-DN separately as mentioned in the Figure. After 24 h of transfection cells were treated with 1 µM plumbagin (Sigma, USA) for 12 h in culture and then assayed for apoptosis using the Annexin-V-PE & 7-AAD apoptosis detection kit (BD Biosciences, USA). For annexin-V assay after staining with Annexin-V-PE &7-AAD the cells were subjected to flow cytometry analysis to detect the externalization of phosphatidyl serine (PS) on cell membrane, a characteristic feature of apoptosis using FACS calibur (BD Biosciences, USA). Apoptosis was also detected by using the TUNEL assay for which after 12 h of treatment with plumbagin, cells were harvested, fixed with 1% paraformaldehyde (Sigma, USA) followed by washing with PBS and storage in 70% ethanol at −20°C. After 18 hr of storage at −20°C the cells were washed with PBS and subjected to TUNEL staining using the APO Direct Kit supplied by BD Biosciences and analyzed by flow cytometer.

## Results

### EVI1 binds to Bcl-xL promoter both *in vitro* and *in vivo* through the 1^st^ set of zinc finger

Although several authors have shown evasion of apoptosis in both EVI1 positive hematopoietic and epithelial cells suggesting that EVI1 is a survival factor, none of them have shown a direct relation between EVI1 and any of the apoptosis regulators. To know if EVI1 directly regulates apoptosis we bioinformatically (www.cbrc.jp/research/db/TFSEARCH.html) scanned a set of both pro-and anti- apoptotic reference gene sequences for EVI1 binding sites and found that Bcl-xL promoter contains a characteristic motif for the binding of EVI1. A sequence of “TTATCTTGGCT” in the non-coding region of 2^nd^ exon, 113 bp upstream of the start codon showed a score of 89 (default score is 85). Also inspection of the same sequence revealed a similar result with MATINSPECTOR (0.89) (www.genomatix.de) and TRANSFAC (0.886) (www.biobase-international.com). Most interestingly the same region was previously shown to be a target of GATA1 and EVI1 by computational analysis in mouse Bcl-xL promoter [22]. Studies have shown earlier that the homology between rat, mouse and human Bcl-xL gene is extremely high across the region encompassing exons 1B through to exon 3 [23]. We also found that the identified region is almost conserved across species (Figure 1A). This finding suggests that Bcl-xL could be a direct transcriptional target of EVI1 which is conserved among species. To examine if EVI1 can bind to the site of the Bcl-xL, we performed a gel shift assay using a radio labeled DNA probe covering the region from −85 to −120 with respect to the start codon of the Bcl-xL sequence. The incubation of this probe with nuclear extracts from 293T cells ectopically expressing EVI1 showed a DNA-protein complex (Figure 1B, lane 1) while no such binding was observed with only 293T cells (lane 3). The complex was not observed with a mutated Bcl-xL oligomer (lane 2). Increasing amount of specific cold competitor at 30× molar excess of radiolabelled probe failed to inhibit the binding of EVI1 to Bcl-xL promoter (lane 4) however a cold competitor at 50× molar excess of radiolabelled probe inhibited the binding of EVI1 to Bcl-xL promoter probe (lane 5). Thus we conclude that EVI1 binds *in vitro* to the Bcl-xL promoter.

To determine whether EVI1 binds directly with this region *in vivo*, we used chromatin immunoprecipitation (ChIP) assay in HT-29 cells, a colon carcinoma cell line, which expresses EVI1. After cross linking and chromatin fractionations the DNA-protein complex was immunoprecipitated with EVI1 antibody (Santa Cruz biotechnology, USA) and control IgG. DNA-protein complexes were processed as per the manufacturer's instructions (Imgenex India Pvt. Ltd., India). Eluted DNA samples were then analyzed by PCR using both a set of specific primers flanking the binding region and also a set of non-specific primers that was selected from a region upstream of the binding site. We observed occupancy of EVI1 at the Bcl-xL promoter, whereas no signal was amplified from the IgG immunoprecipitated negative control. No signal was also observed when primers (EVI1-W) from another region were selected that does not contain a putative EVI1 binding sequence (Figure 1C). EVI1 contains two set of zinc finger domains and to understand which set of zinc finger actually binds to the Bcl-xL promoter we transfected either empty vector or EVI1-wt or EVI1-ΔA or EVI1-ΔD separately in 293T cells and checked them in ChIP assay. It was observed that EVI1-wt and EVI1-ΔD binds to Bcl-xL whereas EVI1-ΔA failed to bind the same region (Figure 1D). Thus, EVI1 binds to the sequence identified in the Bcl-xL promoter both *in vitro* and *in vivo* and Bcl-xL joins a group of genes whose promoter is targeted by EVI1.

### Binding of EVI1 up regulates Bcl-xL transcriptional activity

**Figure 2:**
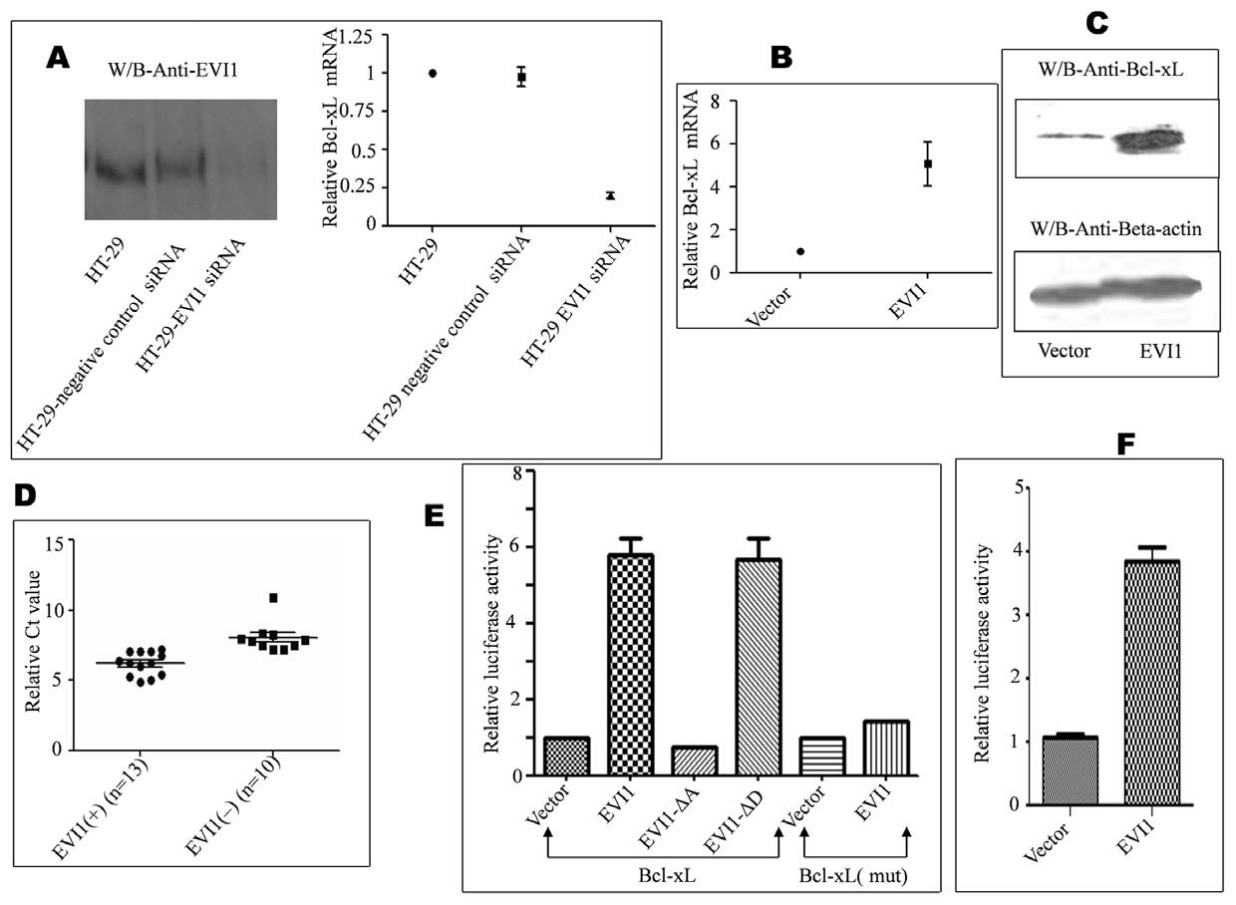
EVI1 induces Bcl-xL promoter trans-activity. A. HT-29 cell line was transfected with EVI1 siRNA and its negative control. After 24 h of transfection total RNA was isolated by TRIzol reagent (Invitrogen). Complementary DNA (cDNA) was prepared using oligodT (Fermentas, Germany) and subsequently RQ-PCR was performed with primers Bcl-xL-F-GCCACTTACCTGAATGACCAC, Bcl-xL-R-TGGATGGTCAGTGTCTGGT with SYBR green gold real time master mix (Eurogentec, Belgium). β-actin primers were used on the same sample for normalization. Bcl-xL level went down by 5.02 fold for siRNA transfected cells as compared to normal cells (p value = 0.0141). The left panel showed the expression of EVI1 protein level. siRNA negative control neither showed any change in Bcl-xL mRNA level nor in EVI1 protein level as compared to only HT-29 cells. B. Bcl-xL mRNA was up regulated in EVI1 transfected cells. 293T cells transfected with only empty vector or EVI1 were processed as described above. The EVI1-positive cells showed 5.07 fold more Bcl-xL expression in comparison to normalized vector control (p value = 0.047). C. Total protein was isolated from 293T cells transfected with only vector or with EVI1 and checked for BCL-XL protein level by standard Western blotting by using anti-Bcl-xL antibody (Imgenex India Pvt. Ltd). D. EVI1 positive (n = 13) and negative cases (n = 10) of CML patients were checked for Bcl-xL mRNA by RQ-PCR as described above. The EVI1 positive cases showed an up regulation of Bcl-xL compared to EVI1 negative cases. (P value = 0.0001). E. 293T cell line was transfected with either empty vector or EVI1 along with the pGL2-Bcl-xL promoter or pGL2 Bcl-xL (mut) and renilla luciferase (internal control). Twenty four hours later the cells were lysed and luciferase activity was measured using the luminometer (GLOMAX 20/20, Promega). EVI1 up regulated the Bcl-xL promoter activity by 5.81 fold and not the mutated promoter. Also EVI1-ΔD up regulated the wild type promoter by 5.68 fold and not EVI1-ΔA. The results were expressed as mean ± s.e. of triplicate experiments, which are representative of three different assays (p value<0.0001). F. NIH3T3 cell line was transfected with either empty vector or EVI1 along with the pGL2-Bcl-xL promoter and renilla luciferase (internal control). Luciferase activity was measured as described above. EVI1 up regulated the Bcl-xL promoter activity by 3.6 fold in NIH3T3 cells (p value = 0.0066).

To accurately quantify the Bcl-xL expression we used real-time PCR assay with RNA extracted from HT-29 cells and also HT-29 cells transfected with a specific EVI1 siRNA and a negative control. Considerable down regulation of EVI1 expression was observed in HT-29 cells treated with EVI1 siRNA (Figure 2A-left panel). By down regulating EVI1 by specific EVI1 siRNA we found that Bcl-xL was also down regulated by almost 5 times (Figure 2A). Negative control siRNA transfected cells didn't show any change. The data was normalized to the endogenous expression of β-actin. We also found that Bcl-xL was up regulated almost 5.07 fold in EVI1 transfected 293T cells with respect to normalized vector control (Figure 2B). This up regulation of Bcl-xL mRNA was also reflected in the levels of BCL-XL protein expression (Figure 2C). Our laboratory works on CML disease progression using CML patient samples (with approval from human ethical committee). As EVI1 is known to be up regulated in CML disease progression we tested all our samples for EVI1 positivity. In this study we statistically showed that Bcl-xL transcriptional activity is different in CML samples depending on the presence or absence of EVI1. EVI1 positive samples showed a significantly more Bcl-xL expression than the EVI1 negative samples (Figure 2D).

To further clarify the transcriptional regulation of EVI1 binding to Bcl-xL, we did a luciferase assay using a mouse Bcl-xL reporter construct, pGL2-Bcl-xL (kind gift from Gabriel Nunez, University of Michigan Medical School, Ann Arbor, Michigan) which covers 3.2 kb upstream from the Bcl-xL start codon in both 293T and NIH3T3 cells. It was already reported earlier that the EVI1 binding site is same in human and mouse (Figure 1A). The EVI1 transfected cells showed a significantly higher luciferase activity (5.81 fold) than the vector transfected cells (Figure 2E, 2^nd^ bar). Mutation in the EVI1 binding region failed to activate the promoter (Figure 2E, bar 6 with respect to bar 5). The assay result is a mean of three independent experiments. We also found a significant increase in the activity with EVI1-ΔD where as no such change was observed with EVI1-ΔA (Figure 2E, bar 3 and 4) hence corroborating the ChIP data that the first set of zinc finger binds to Bcl-xL promoter and up regulates its activity. Bioinformatics analysis has earlier shown that the same site is also occupied by the GATA1 transcription factor [22]. The role of GATA1 in Bcl-xL expression has been obscure because mutations in the typical GATA binding site did not affect GATA1 activated Bcl-xL transcription [24]. However contrasting report states that GATA1 binds to the GATT motif (404 bp or 408 bp upstream of the translation start site in mouse and human Bcl-xL promoters) and mutation of the site shows deregulation of Bcl-xL function [25]. As mutation in the GATA1 binding site (408 bp upstream of the translation start site) down regulates Bcl-xL so the bioinformatically gathered GATA1 site [22] 113 bp upstream of the translation start site may not be active. It is possible that EVI1 directly binds to the region 113 bp upstream of the start codon and GATA1 binds to EVI1 at this site to give a delayed kinetics of Bcl-xL induction as it has been shown that both the proteins interact with each other [26]. Again to rule out the possibility that GATA1 binds to the same region we also used NIH 3T3 cells which lack GATA1 to show that EVI1 can directly up regulate Bcl-xL promoter activity (Figure 2F). Thus all of the above shows that EVI1 directly binds to the Bcl-xL promoter through its proximal set of zinc finger and in the process can regulate its activity.

### Acetylated EVI1 abrogates Bcl-xL promoter binding and down regulates its transcriptional activity

**Figure 3:**
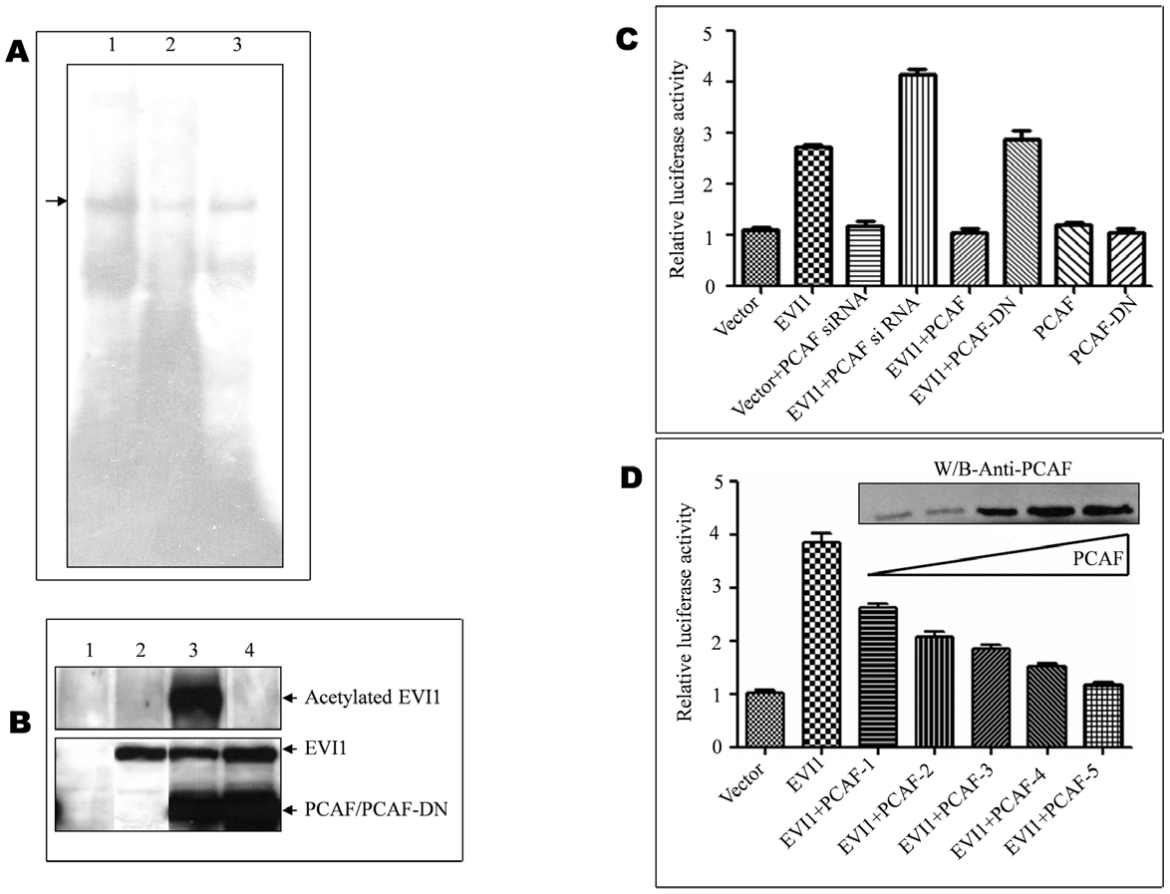
Acetylated EVI1 deregulates Bcl-xL binding and transactivation. A. Nuclear extracts from EVI1-wt or EVI1-wt+PCAF-wt or EVI1-wt+PCAF-DN transfected cells were incubated with a radiolabelled, double-stranded oligo containing the wild type Bcl-xL EVI1 site and analyzed by EMSA. EVI1 binds to Bcl-xL *in vitro* (as shown earlier) (lane 1). However, acetylated EVI1 (EVI1+PCAF-wt) failed to bind the Bcl-xL probe (lane 2). The binding was observed again when EVI1 was transfected along with PCAF-DN (lane 3). B. The same nuclear extract was electrophoresed on a SDS-PAGE and transferred to a PVDF membrane which was subsequently probed with the anti acetylated lysine antibody (Cell Signaling Technology, USA). Acetylated band of EVI1 was observed only when EVI1 was transfected along with PCAF (upper panel, lane 3) whereas no acetylated band was observed when EVI1 was transfected alone (upper panel, lane 2) or with PCAF-DN (upper panel, lane 4). The same blot was stripped and reprobed with the anti-flag antibody (Sigma). Expression of EVI1, PCAF and PCAF-DN was observed as shown on the figure (lower panel). Lane 1 showed the only empty vector transfected nuclear extract. C. 293T cells were transfected with constructs as mentioned in the figure along with the promoter construct and renilla luciferase (as an internal control). Twenty four hours later the cells were lysed and luciferase activity was measured using the luminometer. Up regulation of Bcl-xL was blocked when cells were transfected with both EVI1 and PCAF-wt; however the block was released when cells were transfected with EVI1-wt and PCAF-DN. Only PCAF-wt and PCAF-DN showed no effect on the promoter activity. The respective fold change with respect to vector for EVI1-wt, Vector with PCAF siRNA, EVI1 with PCAF siRNA, EVI1-wt with PCAF-wt, EVI1-wt with PCAF-DN, PCAF-wt and PCAF-DN are 2.72, 1.18, 4.14, 1.052, 2.89, 1.20 and 1.046 (p value<0.0001). D. 293T cells were transfected with vector and EVI1 with increasing concentrations of PCAF (1 µg to 5 µg) with the promoter construct and renilla luciferase. Luciferase assay was done as described above. Up regulation of Bcl-xL was blocked by PCAF in a dose dependent manner (p value<0.0001).

Posttranslational-modifications, mainly acetylation of non-histone proteins show's differential transcriptional activity, including both positive and negative effects on DNA binding affinity. Earlier we have shown that the proto-oncogene EVI1 was acetylated in vivo [19]. Since studies have shown that reversible acetylation of EVI1 are associated with nuclear speckles and nuclear speckles are known to be associated with different cellular functions including apoptosis we wanted to know if acetylated EVI1 has any role on Bcl-xL DNA binding, transcriptional activity and eventually apoptosis. Here we found by EMSA that the incubation of the Bcl-xL probe with nuclear extracts from 293T cells transfected with both EVI1 and PCAF abolished EVI1 DNA binding (Figure 3A, lane 2) with respect to nuclear extracts from cells transfected with EVI1 only (Figure 3A, lane 1). To understand whether protein-protein interaction between EVI1 and PCAF abolishes the binding of EVI1/PCAF complex to Bcl-xL or it is acetylation of EVI1 by PCAF that is abolishing the binding of the complex we used a dominant negative form of PCAF (PCAF-DN). It was shown earlier that a dominant negative form of PCAF fails to acetylate either histones or other proteins. Interestingly the DNA binding was again observed when nuclear extracts from cells transfected with EVI1 and PCAF-DN was used (Figure 3A, lane 3). The acetylation/deacetylation pattern of EVI1 with PCAF or PCAF-DN was shown by Western blot using an anti-acetyl lysine antibody (Figure 3B, upper panel). The same blot was stripped and reprobed with anti-flag antibody to show the expression of EVI1 and PCAF/PCAF-DN (Figure 3B, lower panel). We also wanted to know if acetylation/deacetylation of EVI1 and the reduced binding to Bcl-xL also affects the transcriptional activity. Unlike the previous work where acetylated EVI1 activated the synthetic promoter [19] here we found that PCAF acetylated EVI1 reduced Bcl-xL transcriptional activity with respect to wild type EVI1 in 293T cells (Figure 3C). However, when PCAF-DN was used with EVI1, the Bcl-xL transcriptional activity was higher (almost identical to wild type EVI1 alone) (Figure 3C). PCAF and PCAF-DN didn't show any activity on the Bcl-xL promoter element. To check whether endogenous PCAF has any role, PCAF siRNA was used and a further up regulation of the Bcl-xL promoter element was observed which proves that PCAF mediated acetylation of EVI1 down regulates Bcl-xL transcriptional activity (Figure 3C). The above result was substantiated when increasing concentration of PCAF dramatically decreased the EVI1 mediated up regulation in a dose dependent manner (Figure 3D). Thus acetylation can regulate EVI1 downstream gene transcription in cells by blocking EVI1-Bcl-xL DNA binding activity.

### Acetylation of EVI1 induces apoptosis in HT-29 cells

**Figure 4:**
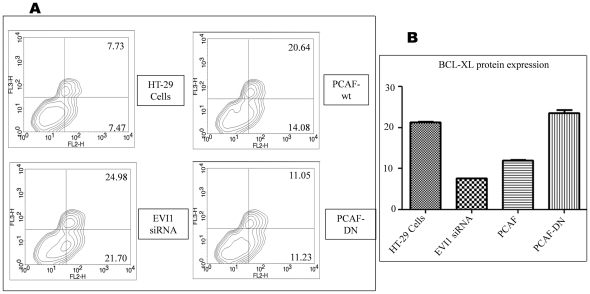
Acetylation of EVI1 induces apoptosis in HT-29 cells (Annexin-V-binding assay). A. HT-29 cells transfected with EVI1 specific siRNA or PCAF-wt or PCAF-DN were cultured for twenty four hours and then 1 µM of plumbagin (Sigma) was added for another twelve hours. After thirty six hours of transfection, surface exposure of phosphatidyl serine in the apoptotic cells was measured by Annexin-V-PE and 7-AAD, by using the Annexin-V-PE apoptosis detection kit (BD biosciences, USA) as per the instruction of the manufacturer. Apoptotic cells are Annexin-V positive and 7 AAD negative but dead cells are positive for both. The percentages of cells that are positive for Annexin-V are 15.2, 46.68, 34.72 and 22.28 for HT-29 cells, EVI1 specific siRNA transfected HT-29 cells, PCAF-wt or PCAF-DN transfected HT-29 cells respectively. B. A part of the same transfected cells that were used for apoptosis assay was checked for the BCL-XL protein expression with Bcl-xL antibody (Imgenex India Pvt. Ltd.) through fluorescence intensity by FACS. As shown BCL-XL expression was higher in HT-29 cells however BCL-XL expression was reduced when EVI1 siRNA or PCAF was transfected in the HT-29 cells. Expression of PCAF-DN again increased the BCL-XL expression (p value<0.0001).

EVI1 blocks cell death by selectively binding and inhibiting JNK and also EVI1 specifically represses the IFN-dependent induction of the tumor suppressor PML and blocks the apoptotic pathway activated by PML [21], [27]. In addition to the ability to inhibit Smad-mediated signaling, EVI1 also functions as a survival gene by activating an anti-apoptotic P13K/AKT signaling pathway in both non-transformed intestinal epithelial cells and in colon cancer cells. To examine the potential role of EVI1 in salvaging the cells from apoptotic death, by external stimuli, we performed apoptosis assay in HT-29 cells. Earlier apoptosis assay was shown in HT-29 cells by using taxol [8], however we have used plumbagin as plumbagin has no effect on PCAF mediated acetylation *in vivo* [28]. EVI1 siRNA was used for a set of cells to down regulate the expression of EVI1. Cells were treated in duplicate with plumbagin for 12 h in culture and then assayed for apoptosis using the Annexin-V-PE & 7-AAD apoptosis detection kit. After staining with Annexin-V-PE & 7-AAD the cells were subjected to flow cytometry analysis to detect the externalization of phosphatidyl serine (PS) on cell membrane, a characteristic feature of apoptosis. As shown in Figure 4A, HT-29 cells showed reduced apoptosis with respect to cells that express the EVI1 siRNA suggesting a direct protective role of EVI1 from apoptotic death of cells. It is becoming increasingly apparent that acetyltransferases act as mediators of environmental signals that can dictate the commitment to cell growth, differentiation or apoptosis. Here we found that acetylation of EVI1 by PCAF increased apoptosis which was again decreased when PCAF-DN was used confirming that here acetylated EVI1 failed to bind Bcl-xL and hence was not able to up regulate its activity (Figure 4A). As noted earlier in Figure 2C all of the transfection showed a substantial change in the regulation of BCL-XL protein expression (Figure 4B) confirming that EVI1 is directly regulating Bcl-xL.

**Figure 5:**
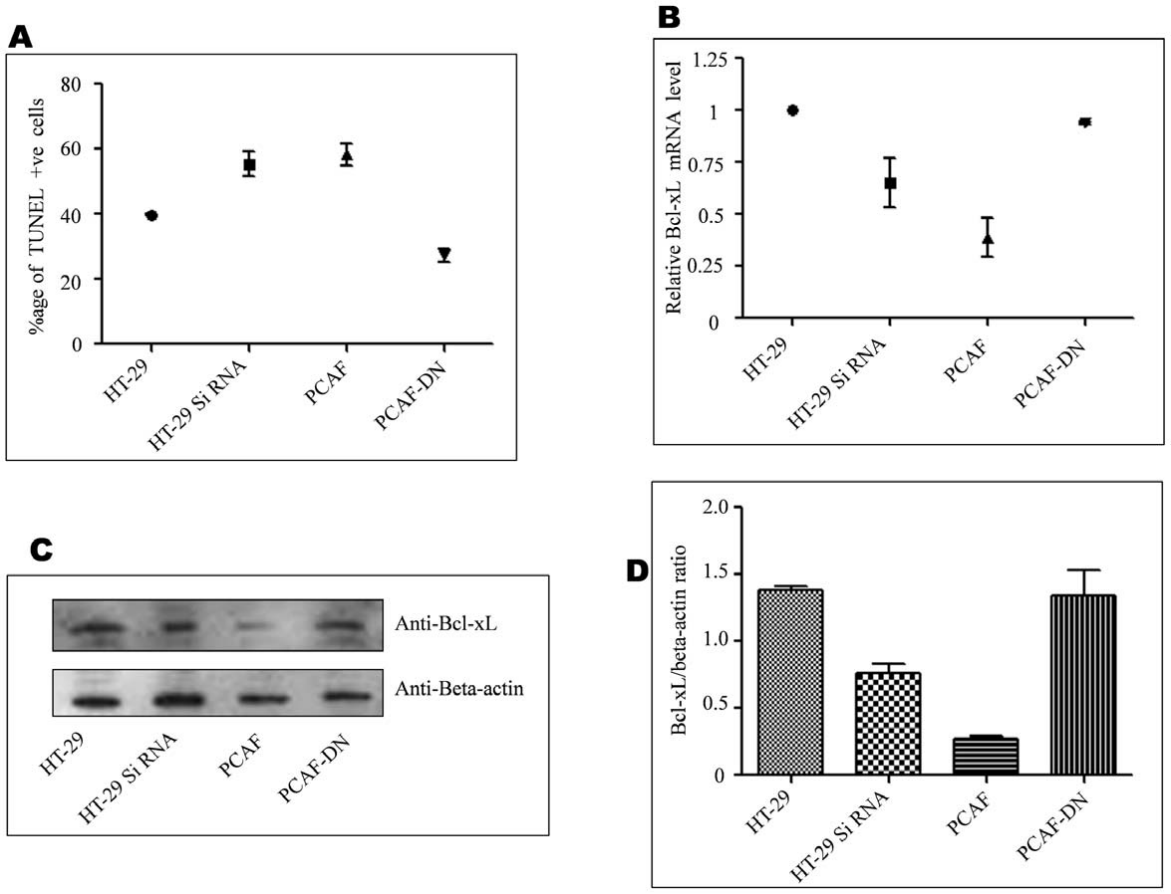
Acetylation of EVI1 induces apoptosis in HT-29 cells (TUNEL assay). A. HT-29 cells were transfected and treated as above (Figure 4). After thirty six hours of transfection, cells were stained with TUNEL reagent (BD biosciences, USA) as per the instruction of the manufacturer. The approximate percentages of cells that are positive for TUNEL are 41, 55, 58 and 28 for HT-29 cells, EVI1 specific siRNA transfected HT-29 cells, PCAF-wt or PCAF-DN transfected HT-29 cells respectively (p value = 0.0036). B. A part of the same transfected cells that were used for apoptosis assay was checked for the BCL-XL mRNA level through real time PCR. As shown Bcl-xL mRNA level was higher in HT-29 cells however it was reduced when EVI1 siRNA or PCAF was transfected in the HT-29 cells. Expression of PCAF-DN again increased the Bcl-xL mRNA level (p value = 0.0016). C. Another part of the same transfected cells that were used for apoptosis assay was checked for the BCL-XL protein expression with Bcl-xL antibody (Imgenex India Pvt. Ltd.) and beta-actin antibody (Santa Cruz) through western blotting. As shown BCL-XL expression was higher in HT-29 cells however BCL-XL expression was reduced when EVI1 siRNA or PCAF was transfected in the HT-29 cells. Expression of PCAF-DN again increased the BCL-XL expression. D. The same western blotting data was quantified by Bio-rad gel doc volume analysis method. Figure shows a quantification of Bcl-xL with respect to the beta actin (p value = 0.0039).

The same transfections were carried out again and after treatment with plumbagin cells were stained with TUNEL reagent and assayed with flow cytometry. As shown in Figure 5A apoptosis increased when cells were transfected with EVI1 siRNA or PCAF while PCAF-DN showed no effect. The same cells were analyzed for Bcl-xL both at transcript level (Figure 5B) and protein level (Figure 5C). Figure 5B showed that Bcl-xL activity went down when HT-29 cells were transfected with either EVI1 siRNA or PCAF. Also the same trend was seen in the protein level of BCL-XL (Figure 5C). The western blotting data was quantified by using Bio-rad gel documentation system volume analysis method for the quantification of Bcl-xL with respect to the beta actin (Figure 5D).

## Discussion

Apoptosis, triggered by a variety of intra- and extra cellular signals, is important for normal development, to maintain tissue homeostasis, and as a defense strategy against the emergence of cancer [29], [30]. However, alterations in the relative level of intracellular pro- *versus* anti-apoptotic Bcl-2 family proteins are implicated in tumorigenesis in both clinical cases and transgenic models [31]. Clinical studies that demonstrated increased level of Bcl-xL in breast carcinoma, gastric cancer cells and others are associated with a poor outcome. In addition, these members are also important determinants of anticancer drug sensitivity [32]. We found that the proto-oncogene EVI1 not only binds Bcl-xL promoter but also up regulates its transcriptional activity and protein expression. We confirmed the occupancy of this site by EVI1 using ChIP analysis in HT-29/293T cells and also by EMSA in 293T cells transfected with EVI1. This is an important finding as other than EVI1 mediated GATA-2 and Pbx1 activation, the proto oncogene has been shown to act as a repressor. Bcl-xL on the other hand is regulated by several other transcription factor families, including STATs (signal transducers and activators of transcription) [33], NF-κB [34], Ets [35], GATA [24], and HIF-1α [17], suggesting that Bcl-xL is induced by different transcription mechanisms depending on the cellular context and the stimulated signals. It is interesting to note that all of the above factors are normally expressed in one or the other cell system; however EVI1 is inappropriately up regulated during oncogenesis. Cells over expressing Bcl-xL mediated by an oncogene can help in the cell survival following DNA damage and can potentially accumulate new somatic mutations at higher frequencies [36]. It was reported earlier that activation of STAT5 can contribute to Bcl-X induction in CML; however STAT5 by itself is not sufficient. Other pathways including activation through IL-3 receptor or PI-3K has also been reported. Thus in an advanced CML case it may so happen that EVI1 acts along with others for maximum accumulation of BCL-XL protein and thus blocks apoptosis. The resulting persistence accumulation and mutations could lead to progression of disease as has been seen in chronic myeloid leukemia, where EVI1 synergizes with the pre-existing oncogene (such as BCR-ABL) to give a highly proliferating and aggressive leukemic clone in a subgroup of CML patients that progresses to blast crisis. Thus, clearly it will be of interest to understand how the expression of Bcl-xL can be regulated in response to the proto-oncogene EVI1 for effective therapeutic intervention.

An important outcome of our study is the provision of a mechanism for the role of acetylation in regulating EVI1 activity. Although all of the transcription factors that bind to Bcl-xL are acetylated, nothing is known how their acetylated form regulates Bcl-xL. Acetylation by lysine acetyltransferases like CBP/p300, PCAF, MYST family modulate the activity of many genes by modifying the lysine residues of both core histones and transcription-related factors and thus play an important role in tumor suppression, apoptosis, cellular proliferation. CBP and PCAF associate with Ku70 *in vivo* and inhibit the ability of Ku70 to suppress Bax-mediated apoptosis, demonstrating that acetylation negatively regulates the antiapoptotic function of Ku70 in vivo, thus providing a critical link between acetyltransferase and tumor suppression [37]. On a separate note, acetylation of FoxO1 activates Bim expression to induce apoptosis in response to HDAC inhibitor depsipeptide [38], but FoxO3 induced expression of the cell death gene BIM was not inhibited by treatment of cells with nicotinamide and TSA [39]. MYST family mediated acetylation deficient p53 fails to induce apoptosis but not cell cycle arrest as it can selectively block the transcription of proapoptotic target genes such as BAX and PUMA while the nonapoptotic targets p21 and hMDM2 remain unaffected [40]. Any deregulation of acetylation/deacetylation equilibrium or inappropriate modifications could lead to different diseases. Previous studies have shown that reversible acetylation of EVI1 is associated with nuclear speckles that are known to be associated with different cellular functions including apoptosis. Here we show decreased transcription of antiapoptotic targets by acetylated EVI1. The sequence-specific DNA binding activity of EVI1 was shown to be dramatically affected by acetylation like in HSF1 and IRF7 [41], [42], possibly as a result of an acetylation induced change in charge. Acetylation can bring about a change in the apoptosis network by reversing the apoptosis block induced by EVI1. Additional experiments will also be needed to determine what signals and proteins promote the activity of PCAF under anti-/proapoptotic conditions. Our data clearly shows that acetylated and deacetylated EVI1 divergently regulates the apoptotic pathway by influencing Bcl-xL. This is very interesting as ineffective hematopoiesis resulting in peripheral blood cytopenias is a hallmark of MDS (EVI1 is reported in both MDS and MDS-AML), and excessive apoptosis of hematopoietic precursors in the marrow appears to be one of the underlying mechanisms. It is not well understood how a disease characterized by excessive apoptosis transforms into one with considerable apoptosis resistance. Elucidation of EVI1 dependent Bcl-xL expression may provide a new dimension for understanding MDS disease progression as disruption of apoptosis pathways may also allow for damaged cells to survive and acquire the characteristics of transformed cells. Our results are especially attractive because it could lead to the development of new therapeutic drugs that can selectively enhance the enzymatic activity of specific acetyltransferases that are involved in acetylation of EVI1. More extensive analysis is underway to elucidate additional functions of EVI1 that may be regulated by acetylation.
